# Exosomes in liquid biopsy and oncology: Nanotechnological interplay and the quest to overcome cancer drug resistance

**DOI:** 10.1016/j.jlb.2023.100134

**Published:** 2023-12-16

**Authors:** Nobendu Mukerjee, Hanan M. Alharbi, Swastika Maitra, Krishnan Anand, Nanasaheb Thorat, Sukhamoy Gorai

**Affiliations:** a Center for Global Health Research, Saveetha Medical College and Hospital, Saveetha Institute of Medical and Technical Sciences, Chennai, India; b Department of Microbiology, West Bengal State University, Barasat, Kolkata- 700126, India; c Department of Pharmaceutics, College of Pharmacy, Umm Al-Qura University, Makkah, 21955, Saudi Arabia; d Department of Microbiology, Adamas University, Barasat, Kolkata, 700126, India; e Department of Chemical Pathology, School of Pathology, Faculty of Health Sciences, University of the Free State, Bloemfontein, 9300, South Africa; f Limerick Digital Cancer Research Centre and Department of Physics, Bernal Institute, University of Limerick, Castletroy, Co. Limerick, Limerick, V94T9PX, Ireland; g Department of Neurological Sciences, Rush University Medical Center, Chicago, IL, USA

**Keywords:** Exosomes, Liquid biopsy, Cancer drug resistance, Nanotechnology, Precision medicine

## Abstract

Exosomes, small extracellular vesicles of endocytic origin, have emerged as pivotal mediators in intercellular communication, driving transformative advancements across diverse fields of biology and medicine. This comprehensive review delves into the multifaceted roles of exosomes in health and disease, elucidating their biogenesis, cargo composition, and far-reaching implications. Exosomes, secreted by virtually all cell types, encapsulate a cargo comprising proteins, lipids, and nucleic acids, reflecting their cellular origin. Their molecular cargo modulates cellular processes, facilitating complex signalling cascades and contributing to the pathogenesis of various diseases, including cancer, neurodegenerative disorders, and infectious diseases. In cancer, exosomes serve as messengers of tumorigenesis and metastasis, orchestrating critical events within the tumor microenvironment. Furthermore, exosomes participate in drug resistance mechanisms, presenting significant challenges in cancer therapy. The diagnostic potential of exosomes, particularly in the context of liquid biopsy, is underscored by their presence in various biofluids. This offers non-invasive disease monitoring and biomarker discovery, revolutionizing early detection and monitoring strategies. Additionally, exosomes have gained recognition as therapeutic vehicles, holding promise for targeted drug delivery, immunomodulation, and regenerative medicine. This review comprehensively explores the ever-expanding landscape of exosome biology, emphasizing their roles in health and disease. It underscores the transformative potential of exosomes in liquid biopsy-based diagnostics and therapeutics while acknowledging the complexities and challenges that lie ahead in harnessing their full clinical utility.

## 1 Introduction

Extracellular vehicles (EVs), encompassing exosomes, microvesicles, and apoptotic bodies, are commanding increasing attention within the realm of cellular biology, with a particular focus on their critical roles in various scientific endeavours, notably in the field of cancer biology. These nanosized vesicles, which range in size from 30 to 150 nm for exosomes to larger dimensions for microvesicles and apoptotic bodies, have transcended their initial perception as mere cellular byproducts. Instead, they are now recognized as sophisticated vehicles of intercellular communication, ferrying a diverse array of biological messages across various cellular environments [1]. Originating from different cellular compartments and released by a multitude of cell types, EVs carry a distinctive molecular signature. They house a diverse cargo comprising proteins, lipids, mRNAs, and microRNAs, serving as a reflective snapshot of their parent cell's physiological state. Consequently, they play pivotal roles in both health and disease, with the potential to serve as biomarkers and influence recipient cell phenotypes, thereby modulating a myriad of biological processes [2]. In the intricate landscape of cancer biology, the significance of EVs, especially exosomes, is underscored further. They have been implicated in numerous oncogenic processes, ranging from promoting tumor cell proliferation, migration, and invasion to orchestrating angiogenesis and metastasis. More alarmingly, EVs have emerged as critical contributors to drug resistance, a major hurdle in successful cancer therapeutics [3]. The complex interplay between EVs and the tumor microenvironment (TME) weaves a web of interactions that can fuel tumor growth, shape immune responses, and contribute to therapeutic failures [4]. Concurrently, the intersection of EV biology with nanotechnology promises groundbreaking innovations. Due to their nanoscale size, biocompatibility, and ability to traverse biological barriers, EVs, particularly exosomes, are undergoing rigorous investigation as natural nanocarriers for targeted drug delivery. Their inherent potential for engineering further accentuates their promise in personalized medicine, envisioning a future where cancer therapies are not only targeted but also devoid of off-target side effects [5]. This article embarks on a meticulous exploration of the roles played by EVs, including exosomes, microvesicles, and apoptotic bodies, in the context of cancer. It spans from their fundamental biology to their intricate involvement in oncogenic processes and their prospective nanotechnological applications. Through a detailed lens, we aim to shed light on the vast landscape of EV research, offering insights that might pave the way for groundbreaking advancements in cancer diagnostics, therapeutics, and beyond.

## 2 The biology of exosomes

Exosomes, classified as a subset of extracellular vesicles, emerge from the intricate world of cellular trafficking. They are birthed from endosomal compartments, more specifically from intraluminal vesicles that coalesce to form multivesicular endosomes (MVEs). As these MVEs fuse with the plasma membrane, they release these vesicles into the extracellular milieu, introducing exosomes to their surrounding environment [6]. Molecularly, exosomes are distinct from other cellular components. Their membrane is enriched with specific lipids like sphingomyelin, ceramide, and cholesterol, which not only offers structural integrity but also plays a role in their biogenesis and uptake by recipient cells [7]. Inside this lipid bilayer, they house a diverse cargo, a compendium of proteins, mRNAs, microRNAs, long non-coding RNAs, and even genomic DNA fragments [8]. The precise composition of this cargo is reflective of their cell of origin and its current physiological or pathological state. For instance, tumor-derived exosomes might carry oncogenic receptors, enzymes, and nucleic acid sequences that can modulate the phenotype of recipient cells [9]. This meticulously orchestrated bio-signature of exosomes is what catapults them into the limelight of modern biomedicine. Their ability to ferry molecular information from one cell to other positions them as critical mediators of cell-to-cell communication. But beyond this, the diagnostic treasure they bear—owing to their potential reflection of disease states—has spurred extensive research into leveraging exosomes as liquid biopsies for various ailments, including cancers. Furthermore, their ability to encapsulate and deliver molecules has beckoned the therapeutic arena, exploring their potential as drug delivery vehicles or even as direct therapeutic agents [10].

## 3 Role of exosomes in cancer biology

Exosomes, as mediators of intercellular communication, have a profound impact on the multifaceted processes of cancer biology. These extracellular vesicles wield influence over various stages of tumor progression, from initiation to metastasis, underscoring their centrality in oncogenesis. Firstly, tumor-derived exosomes (TDEs) are enriched with oncogenic cargo [11]. They contain specific proteins, mRNAs, and microRNAs that can drive malignant transformation in recipient cells. For instance, exosomal transfer of mutant forms of certain genes or oncogenic microRNAs can lead to the activation of signalling pathways that favour tumor cell proliferation, survival, and migration [12]. Metastasis, the lethal hallmark of malignancy, is another process where exosomes play a pivotal role. TDEs can prepare distant sites for metastatic colonization, a phenomenon known as the pre-metastatic niche formation. They achieve this by modulating stromal cells, endothelial cells, and even components of the immune system to create an environment conducive for circulating tumor cells to implant and thrive [13]. Furthermore, the cross-talk between exosomes and the TME cannot be overemphasized as complex milieu comprising fibroblasts, immune cells, endothelial cells, and extracellular matrix, plays a significant role in tumor progression and resistance to therapy. Exosomes influence the TME by transferring molecules that can induce angiogenesis, suppress immune responses, and promote the activation of cancer-associated fibroblasts. In turn, these modified components of the TME can further support tumor growth and protection against therapeutic agents [14]. Additionally, exosomes have been found to play roles in modulating immune responses within the TME. For instance, TDEs might carry immune-suppressive molecules, leading to the inhibition of effector T cell functions or the promotion of regulatory T cell activities, thus shielding the tumor from immune surveillance [15,16]. In summary, the involvement of exosomes in cancer biology is profound and multifactorial. Their role as conveyors of oncogenic signals, facilitators of metastasis, modulators of the TME, and manipulators of immune responses positions them at the epicentre of innovative cancer research, offering potential avenues for therapeutic interventions.

## 4 Exosomes and cancer drug resistance

**Fig. 1:**
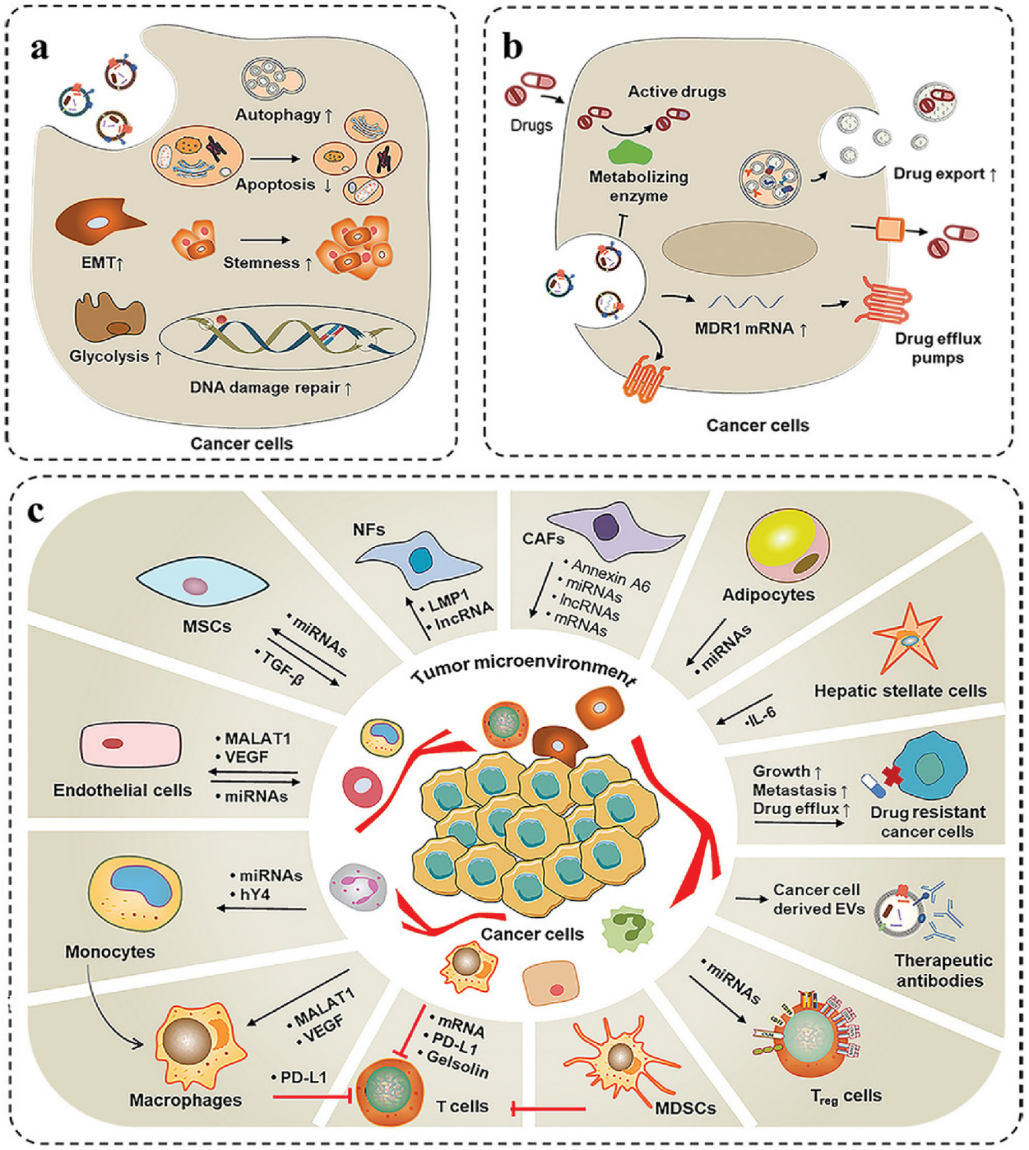
The role and mechanism of EVs in cancer drug resistance. a) EVs transfer specific cargos that regulate signalling pathways associated with autophagy, metabolism, EMT, and CSCs, thus playing important roles in the development of cancer drug resistance. b) EVs contain drug efflux transporters that directly mediate the active extrusion of drugs, leading to the development of drug resistance. c) EVs transport biological active substances to mediate multiple layers of interactions between cancer cells and TME cells, leading to the acquisition of drug resistance. (Reproduced with permission under Creative Commons CC BY 4.0 license from Ref [44], Copyright 2022 The Authors).

Drug resistance in oncology is a formidable barrier that often leads to treatment failure and disease relapse. This resistance is intricately tied to various mechanisms, among which exosomes play a crucial role. Exosomes facilitate both intrinsic and acquired drug resistance in tumor cells, notably through the transport of drug-efflux proteins. For instance, they can carry ATP-binding cassette (ABC) transporters such as P-glycoprotein (P-gp) and multidrug resistance-associated proteins (MRPs), which actively remove chemotherapeutic agents from the cells, thereby diminishing their intracellular concentrations and efficacy [17]. Beyond this active drug removal, the image provided illustrates the broader impact of exosomes on drug resistance. Fig. 1 highlights how exosomes affect cancer cell behaviour and drug resistance through several mechanisms: They alter signalling pathways by transferring molecules like growth factor receptors and kinases, potentially leading to the upregulation of survival and proliferation pathways, such as PI3K/Akt or MAPK, thereby rendering tumor cells less sensitive or even entirely resistant to chemotherapy [18]. Additionally, exosomal miRNAs [19,20] can post-transcriptionally regulate gene expression, targeting proteins crucial to the effectiveness of anti-cancer drugs. By inhibiting pro-apoptotic proteins or enhancing anti-apoptotic ones, these miRNAs contribute to resistance against drugs designed to induce apoptosis in cancer cells [19]. The Fig. 1 further depicts the tumor microenvironment, where exosomes modulate various components such as stromal cells, immune cells, and the extracellular matrix, creating a supportive niche that protects tumor cells from therapeutic agents [21]. This complex interaction, as shown in the image, underlines the significant role of exosomes in drug resistance. Understanding their multifaceted roles is imperative not just for overcoming resistance but also for opening avenues to novel therapeutic strategies. The exosome-mediated communications within the tumor microenvironment, and their role in the resistance to chemotherapy, underscore the potential of exosomal pathways as targets in the development of new cancer treatments.

## 5 TME cells derived exosomes in the regulation of drug resistance

The tumor microenvironment (TME) is a highly intricate and dynamic milieu, composed of a myriad of non-malignant cell types, including cancer-associated fibroblasts (CAFs), immune cells (like tumor-associated macrophages, dendritic cells, and T lymphocytes), endothelial cells, and components of the extracellular matrix. Each of these constituents plays a unique role in sculpting the characteristics and behaviours of resident tumor cells, with profound implications for tumor progression, metastasis, and therapeutic responses [22]. One emerging area of interest is the role of exosomes secreted by TME cells in the regulation of drug resistance. These extracellular vesicles, laden with molecular cargo, act as vital conduits for intercellular communication within the TME. For instance, exosomes derived from CAFs are known to carry a myriad of signaling molecules, growth factors, and miRNAs that can boost the survival and proliferative capacities of tumor cells. These exosomal contents can activate pro-survival pathways in cancer cells or inhibit apoptotic responses, thereby engendering resistance to chemotherapeutic agents [23]. Similarly, immune cell-derived exosomes can also play dual roles. While certain exosomes might have anti-tumor effects, others, especially from immunosuppressive cells like myeloid-derived suppressor cells or regulatory T cells, can carry factors that promote tumor cell growth and therapeutic resistance. These factors might suppress effector immune responses, thereby allowing tumor cells to escape immune surveillance and survive in the presence of therapeutic agents [24]. Furthermore, the interplay between exosomes and components of the extracellular matrix can impact drug resistance. Exosomal proteases or enzymes can remodel the matrix, making it more conducive for tumor cells to thrive and less permeable to drugs. Additionally, exosomal signals can also stimulate angiogenesis, ensuring tumor cells receive adequate nutrients and oxygen, further complicating the therapeutic landscape [25]. The myriad exosomal interactions within the TME highlight the complexity of drug resistance mechanisms in cancer. A deeper comprehension of these interactions not only elucidates the multifactorial nature of drug resistance but also opens the doors to novel therapeutic avenues that target these intricate communication networks.

## 6 The implications of exosomes in monitoring cancer therapy response

Exosomes, minute extracellular vesicles laden with a diverse array of biologically active molecules that faithfully mirror their parent cells, have emerged as focal points of intense scrutiny in the field of cancer diagnostics and prognostics. Their remarkable stability in various bodily fluids, including blood, urine, and cerebrospinal fluid, combined with their inherent capacity to function as natural intercellular shuttles for non-coding RNAs (ncRNAs), such as microRNAs (miRNAs) and circular RNAs (circRNAs), places them in a pivotal role at the forefront of liquid biopsy research for cancer detection and monitoring [26]. Within the context of rapidly advancing nanotechnology, researchers have devised innovative methodologies to isolate and characterize exosomes with heightened sensitivity and specificity. Nano-plasmonic sensors, for instance, exhibit the extraordinary ability to detect exosomal surface markers with exceptional precision. Simultaneously, nanoparticle-based assays have been developed to enable the quantification and profiling of exosomal RNA and protein content in a high-throughput manner, thus offering a deeper understanding of the regulatory effects mediated by exosomal ncRNAs [27,28]. In the continuum of cancer therapy, the dynamic changes observed in the molecular composition of exosomes, particularly concerning their ncRNA cargo, serve as invaluable sources of real-time feedback on therapeutic efficacy. For example, a discernible reduction in exosomal ncRNAs associated with tumor aggressiveness or proliferation may serve as an encouraging indicator of a positive response to therapy. Conversely, the emergence of specific ncRNAs, such as drug-efflux proteins or resistance-associated miRNAs, may serve as early indicators of acquired drug resistance, potentially preceding clinical manifestations of treatment failure [29]. Moreover, within the context of targeted therapies, exosomes have emerged as carriers of specific mutated genes or pathway components, thus acting as informative messengers that facilitate informed treatment decisions. For instance, the presence of exosomal EGFR mutations in patients with non-small cell lung cancer can provide a crucial basis for the utilization of specific EGFR inhibitors, thereby enabling highly personalized and effective treatment strategies [30]. The confluence of exosome biology, specifically their role as carriers of regulatory ncRNAs, and the transformative potential of nanotechnology represents a promising horizon for monitoring cancer therapy responses. By providing real-time, non-invasive insights into tumor dynamics and therapy outcomes, exosomes stand poised to revolutionize cancer management. They facilitate timely interventions and underscore the importance of personalized treatment approaches, marking a significant stride towards more effective and precise cancer care.

## 7 Potential of exosomes in overcoming cancer drug resistance

Exosomes, as nature's endogenous nanocarriers, possess an intriguing potential for reconfiguring the landscape of cancer therapeutics, especially in the realm of drug resistance. Their intrinsic properties, such as stability, biocompatibility, and an innate ability to cross biological barriers, set them apart as unique therapeutic tools [31,32]. In the face of mounting challenges with drug resistance, researchers are exploring ways to manipulate exosomes to achieve therapeutic gains. One avenue is the engineering of exosomes to carry molecular tools that can counteract resistance mechanisms. For example, exosomes can be loaded with siRNAs or miRNAs that target and downregulate drug-efflux proteins or other resistance-associated genes in tumor cells, effectively sensitizing them to chemotherapy [33]. Nanotechnology offers a plethora of opportunities to enhance the therapeutic potential of exosomes. Techniques like electroporation, sonication, or nanoprecipitation allow the precise loading of drugs, proteins, or nucleic acids into exosomes [34]. Furthermore, with advancements in surface modification techniques, exosomes can be tailored to possess specific targeting moieties, ensuring their directed delivery to tumor cells or specific components of the tumor microenvironment, thereby minimizing off-target effects and maximizing therapeutic efficacy [35]. Moreover, bioengineered exosomes can be designed to carry a combination of therapeutic agents and imaging probes, creating theragnostic platforms that enable both therapy and real-time monitoring of drug delivery and tumor response [36]. In essence, the amalgamation of exosome biology with nanotechnology has the potential to reshape the approach towards combatting drug resistance in cancer. By harnessing the natural trafficking routes and targeting abilities of exosomes, and enhancing them with cutting-edge nanotechnological tools, we stand on the cusp of a new era in precision oncology, where drug resistance could become a challenge of the past.

## 8 Perspective and future challenges

The burgeoning field of exosome research, particularly when intertwined with the intricate tapestry of nanotechnology, heralds a paradigm shift in our approach to cancer therapy. However, the journey towards translating this potential into clinical realities is laden with multifaceted challenges and complexities [37]. One primary hurdle lies in the efficient isolation and purification of exosomes. Contemporary methodologies, which range from conventional ultracentrifugation to size-exclusion chromatography, often yield heterogeneous vesicle populations. Integrating cutting-edge nanotechnological tools, such as nanoparticle-assisted capture and nano-filtration techniques, exosomes barcoding [38] holds promise in enhancing the purity and specificity of exosome isolation [39]. Another challenge resides in the comprehensive characterization of exosomal contents. Although high-throughput techniques like mass spectrometry and next-generation sequencing have significantly advanced our comprehension of exosomal cargoes, the dynamic nature of these vesicles mandates a continuous refinement of profiling methods. Nanoscale imaging techniques, such as atomic force microscopy and cryo-electron microscopy, can provide intricate morphological and structural insights into these vesicles, contributing to a more comprehensive understanding [40]. Scalability emerges as a significant impediment, particularly concerning therapeutic applications. Transitioning from benchtop research to large-scale production for clinical trials and eventual therapeutic use demands streamlined protocols that preserve the integrity and functionality of exosomes. Here, nanotechnology, with its advancements in bioreactor designs and microfluidic systems, can play a pivotal role in addressing this challenge [41]. Finally, the biocompatibility, immunogenicity, and potential off-target effects of engineered exosomes necessitate rigorous investigation. A thorough comprehension of the interplay between bioengineered exosomes and the host immune system is imperative to ensure safety and efficacy in therapeutic applications [42,43]. While the horizon of exosome research, especially in conjunction with nanotechnology, appears promising, it is imperative to adopt a holistic and integrative approach to navigate the complexities and challenges that lie ahead. This comprehensive review endeavours to elucidate the scientific intricacies and the potential of exosomes in reshaping the landscape of cancer therapy, offering a hopeful yet balanced perspective.

## Ethics approval and consent to participate

Not applicable.

## Consent for publication

Not applicable.

## Data availability statement

Not applicable.

## Declaration of competing interest

The authors are declaring no conflict of interests.
